# Let’s Talk About Sex: An Acceptable, Appropriate and Feasible Online Sexual Health Educational Video Series for Breast Cancer Patients

**DOI:** 10.21203/rs.3.rs-7023075/v1

**Published:** 2025-08-18

**Authors:** Madeline G. Higgins, Laura Helmkamp, Lauren A. Zimmaro, Sarah E. Leslie, Monica Adams, Sudheer Vemuru, Victoria D. Huynh, Erin Baurle, Laura Bozzuto, Kristin E. Rojas, Helen L. Coons, Ashley Arkema, Sarah Tevis

**Affiliations:** University of Colorado Anschutz Medical Campus; University of Colorado Anschutz Medical Campus; University of Colorado Anschutz Medical Campus; University of Colorado Anschutz Medical Campus; University of Houston; University of Colorado Anschutz Medical Campus; The University of Texas MD Anderson Cancer Center; University of Colorado Anschutz Medical Campus; University of Wisconsin–Madison; University of Miami; Women’s Mental Health Associates; University of Colorado Anschutz Medical Campus; University of Colorado Anschutz Medical Campus

**Keywords:** Breast cancer, sexual health, patient-centered resources, quality of life

## Abstract

**Purpose:**

Sexual health-related side effects during breast cancer treatment are common and distressing but not always expected by patients. We created an online sexual health educational video series to increase patients’ awareness of these issues. In this exploratory study, we aimed to evaluate the acceptability, appropriateness, and feasibility of the video series among newly diagnosed breast cancer patients.

**Methods:**

Eligible and interested patients completed a baseline questionnaire within one month of diagnosis or surgical evaluation. Enrolled participants accessed the online sexual health video series. A follow-up questionnaire evaluating acceptability, appropriateness, and feasibility was administered after 6-months. Descriptive statistics and paired t-tests were utilized.

**Results:**

Twenty-eight participants (January-June 2024) with mean age of 48 years (±8 years) comprised the present analysis. Eighty-six percent were White, 4% Hispanic, 89% straight/heterosexual, 86% married/partnered, and 68% were stage I at diagnosis. Eleven participants (39%) completed the 6-month survey, and nine participants reported watching the online videos. Nearly all completely agreed or agreed that the video series was acceptable (mean 4.1 [±1.0]), appropriate (mean 4.3 [± 0.7]), and feasible (mean 4.4 [±0.7]). Qualitative feedback showed strong endorsement for the video series and offered suggested improvements.

**Conclusion:**

Breast cancer patients lack access to sexual health resources during treatment, but an online educational video series is acceptable, appropriate, and feasible to utilize. This video series fills a unique gap in patient care, and with further iterations and development has the potential to significantly advance the sexual health resources available to breast cancer patients and survivors.

## Introduction

Advances in breast cancer treatment have led to more than 3.5 million breast cancer survivors in the United States, many of whom will experience short- and long-term sexual health side effects because of their treatment [1–3]. Sexual health symptoms after breast cancer treatment are common with 37–78% of survivors experiencing at least one sexual concern and up to 87% reporting low sexual desire [1–6]. Patients may also experience negative body image, infertility, menopausal symptoms, concerns about intimacy, and struggles with dating [5, 7]. These symptoms have meaningful impacts as studies have shown that breast cancer patients with sexual concerns report poorer quality of life [3, 8] and greater psychological distress, including greater depression and anxiety, as compared to those without sexual symptoms [9, 10].

While treatment-related sexual health symptoms are common during breast cancer, many patients report a lack of sexual health-specific education and resources from their oncology team or other medical providers [6]. In fact, women with breast cancer report a strong desire for discussions about sexual health to be initiated by their clinician [11–13] and for the topic of sexual health to be brought up early – and often – throughout their breast cancer journey [6]. Multiple studies demonstrate that patients prefer to receive information through discussions with their providers or from handouts, online websites, and support groups [6, 14]. Additionally, focus groups with breast cancer survivors have identified that patients want a broad range of sexual health education from treatment effects to mitigation strategies to navigating dating and partner intimacy [6]. Unfortunately, studies among breast cancer providers have identified key clinician barriers to discussing sexual health with breast cancer patients including lack of clinic time, inadequate knowledge, and discomfort or embarrassment [15, 16]. Clinicians also face conflicting priorities and often focus on cancer outcomes and treatment rather than discussing topics related to intimacy and sexual health [12]. Overall, sexual health discussions occur in less than half of patient visits [17, 18] and when they do, 28% of patients incorrectly self-report whether or not the conversation happened [18].

While there have been significant scientific and clinical advances in addressing cancer-related sexual issues over the past decade, the need for patient-facing educational programs that are accessible, relevant, and comprehensive remains. Current interventions have features that may limit their ability to provide timely, practical, and appealing sexual health education to breast cancer patients and survivors. For example, many interventions are either clinician-facing and aimed at improving communication [19–21], have a narrow scope and are centered on specific issues such as fertility or menopausal symptoms [6], are one-time programs [22] or are only provided post-treatment and after symptoms arise [23–27]. Together, this limits the ability for women to access sexual health information repeatedly or at different timepoints throughout the course of treatment. As such, there remains a continued need for collaborative, patient-centered approaches to develop and implement acceptable, appropriate, and feasible programs to address cancer-related sexual concerns.

Therefore, we assembled a multidisciplinary team including sexual health experts and breast cancer survivors to develop an online video educational series to use throughout patients’ breast cancer care. The iterative and rigorous user-centered development process has been described by Adams et. al [28]. Overall, the video series consisted of 56 separate, brief videos (1–3 minutes long) that provide sexual health education on a range of topics such as 1) medical and biological (i.e., surgical approaches for treatment, expectation setting, possible changes to physical sensation, effects of treatment related to early menopause and estrogen blockades), 2) emotional, behavioral, and cognitive (i.e., coping with anxiety, mindfulness techniques), 3) patient relationships (i.e., accepting support, building relationships with providers), and 4) intimate relationships (i.e., communicating with partners, body image, dating). The videos show short clips from subject matter experts spanning breast surgery, medical oncology, gynecology, psycho-oncology and sexual medicine, plus personal stories and lived-experiences of breast cancer survivors. Preliminary evaluation among prior focus group participants found that the videos were favorable and understandable, however the acceptability, appropriateness, and feasibility among newly diagnosed breast cancer patients was untested [28].

Exploratory in nature, this pilot study aimed to assess the acceptability, appropriateness, and feasibility of the sexual health video series via validated surveys and open-item feedback responses among a sample of newly diagnosed breast cancer patients. Additionally, we sought to examine preliminary signals of intervention effects by assessing patient-reported sexual function from pre- to post-intervention. Given the proof-of-concept nature of this study [29], no specific hypotheses were made, but we expected the intervention to be perceived as acceptable, appropriate, and feasible among participants.

## Methods

### Study Design and Patient Population

This was a pilot study performed as part of an ongoing, prospective clinical trial (NCT06121258) that included Stage I-III adult female breast cancer patients seen in a comprehensive breast cancer center. This study was approved by the Institutional Review Board (COMIRB #23–1734). Eligible women aged 18–100 years old diagnosed with breast cancer provided informed consent and completed study procedures. Patients with Stage 0 or IV breast cancer, prior cancer history, or prior exposure to anti-hormone therapy, chemotherapy or radiotherapy not related to breast cancer were excluded. The online video series was available in English only during this pilot phase, so only patients who could read or speak in English were included. Male breast cancer patients were excluded due to the low prevalence overall [30]. Eligible patients were identified by a research coordinator and contacted via telephone within one month of their initial multi-disciplinary clinic visit or initial surgical consultation, but prior to initiating treatment, to discuss the study and confirm eligibility. Interested patients were then emailed a consent form and instructions for completing a baseline survey including demographic and recent sexual history.

**Figure 1:**
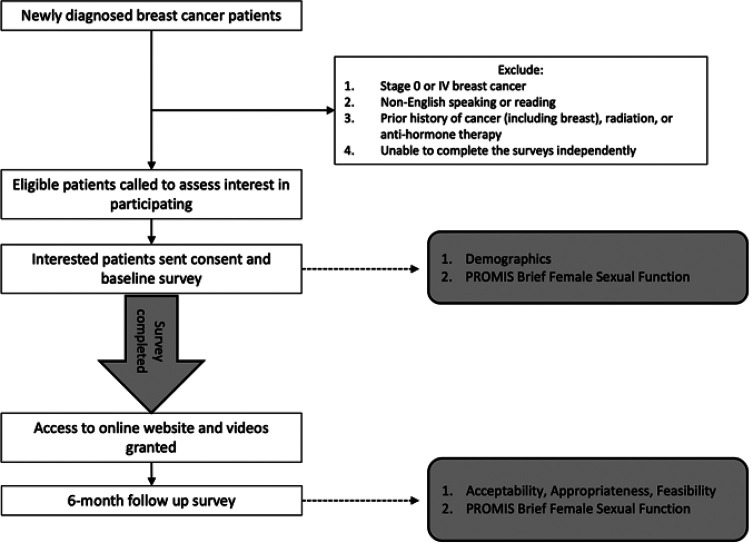
Schematic of study design and measures used.

Following informed consent and completion of the baseline survey, participants were emailed a link to access the online video series website, along with a personalized, de-identified username and password. Due to the individualized nature of breast cancer treatment, participants were provided full access to the entire video series without explicit instructions on the order or selection of videos they should watch. Participants then received a monthly email reminder with a link to the videos for five months and a final survey six months after enrollment. All surveys and reminders were administered electronically via the REDCap Database [31, 32]. Survey reminders were automatically repeated three times, one week apart. After this, patients received up to three individual emails from a study coordinator with a personal link to their six-month survey. No phone calls or attempts to reach the participants in clinic were made. Participants were considered non-responders if they did not respond to the six-month survey within three months. Participants were provided a $25 gift card for completing the baseline survey and an additional $25 gift card for completing the six-month follow-up survey. A schematic outlining the study design is demonstrated in Fig. 1.

### Outcomes

The primary outcomes were acceptability, appropriateness, and feasibility, assessed by the Acceptability of Intervention (AIM), Intervention Appropriateness (IAM), and Feasibility of Intervention Measures (FIM), which are widely used self-reported surveys with good psychometric properties [33]. Responses are measured on a 5-point Likert scale and higher scores reflect a higher degree of acceptability (i.e., is appealing, satisfactory), appropriateness (i.e., is relevant, fitting), and feasibility (i.e., is easy to use, implementable). Participants who did not endorse watching the videos at the six-month survey were excluded from completing the AIM, IAM, and FIM surveys as the purpose was to capture those who watched at least a portion of the videos. The primary outcomes were assessed 6-months postintervention and were chosen to improve study participant retention.

The secondary outcome was the impact of the online educational resource on sexual health, assessed by the NIH-developed PROMIS Brief Female Sexual Function and Satisfaction (FSFS) v2.0 questionnaire at baseline and six-months. The PROMIS Brief FSFS is a validated patient-reported outcome measure that assesses an individual’s sexual function and satisfaction over the past three months in the following subdomains: 1) interest in sexual activity, 2) vaginal lubrication, 3) vaginal discomfort, 4) labial discomfort, 5) clitoral discomfort, 6) orgasm ability, 7) orgasm pleasure, and 8) satisfaction with sex life [34, 35]. This measure was chosen for its greater inclusivity of diverse and varied sexual practices compared to other validated measures of sexual health. In addition to the PROMIS FSFS, a single question was included in the baseline survey assessing sexual activity in the past 6 months prior to the breast cancer diagnosis.

Further exploratory outcomes included evaluation of individual patient perceptions of the experience and impact of the online educational resource on their sexual health during breast cancer treatment. This was assessed through free-response questions throughout the study including a question at 6-months post-intervention, “What did you learn from the videos?”

## Statistical Analysis

Survey responses were collected via REDCap, and clinical data was abstracted through chart review. Participant demographic and clinical factors were described using counts and percentages for categorical variables and means and standard deviations for age. Six-month responders were compared to non-responders using chi-square tests or Fisher’s exact tests as appropriate for categorical variables and a t-test for age. The combined means and SD for the AIM, IAM, and FIM scales were described with values ranging from 1 to 5 with higher scores indicating greater acceptability, appropriateness, or feasibility. The PROMIS FSFS was scored according to the instrument’s scoring manual [36]: Calibrated subdomain scores are expressed in terms of standardized T-scores where a mean score of 50 corresponds with the average response in the reference sample of female respondents (SD = 10). Higher scores indicate greater endorsement of the subdomain assessed (e.g., greater interest in sexual activity, greater vaginal discomfort). Changes in PROMIS scores were calculated by subtracting the baseline score from the 6-month score. Given the exploratory nature of analysis, effects were characterized descriptively via the magnitude of the change score and paired t-tests. Analyses were conducted in SAS version 9.4 with statistical significance set as *p*-value < .05.

## Results

### Study Population

From January – June 2024, 117 patients were screened, and 82 (70%) patients were called for potential inclusion (Supplemental Fig. 1). Of those contacted, 57 (70%) expressed an interest in participating and were emailed the electronic consent and baseline survey. Twenty-eight (49%) patients completed both the consent and baseline survey and were given access to the video series. Of these, 11 (39%) patients completed the 6-month follow up survey, while 17 patients did not complete the 6-month survey.

The mean age of the 28 participants was 47.9 years (SD 8). Most participants were white (85.7%, n = 24), non-Hispanic/Latina or of Spanish origin (96.4%, n = 27), straight/heterosexual (89.3%, n = 25), were married or partnered (85.7%, n = 24), and with a male sexual partner (75.0%, n = 21) (Table 1). The most common sexual activities in the past six months reported by participants were sexual intercourse, oral sex, and masturbation. Clinically, most participants had Stage I disease (67.9%, n = 19) and were diagnosed with invasive ductal carcinoma (82.1%, n = 23) and ER/PR-positive, HER2-negative tumors (75%, n = 21). Additional clinical characteristics are shown in Table 2.

There were no statistically significant differences in baseline demographics and sexual activity among the 6-month responders compared to the non-responders.

### Acceptability, Appropriateness, and Feasibility

**Figure 2:**
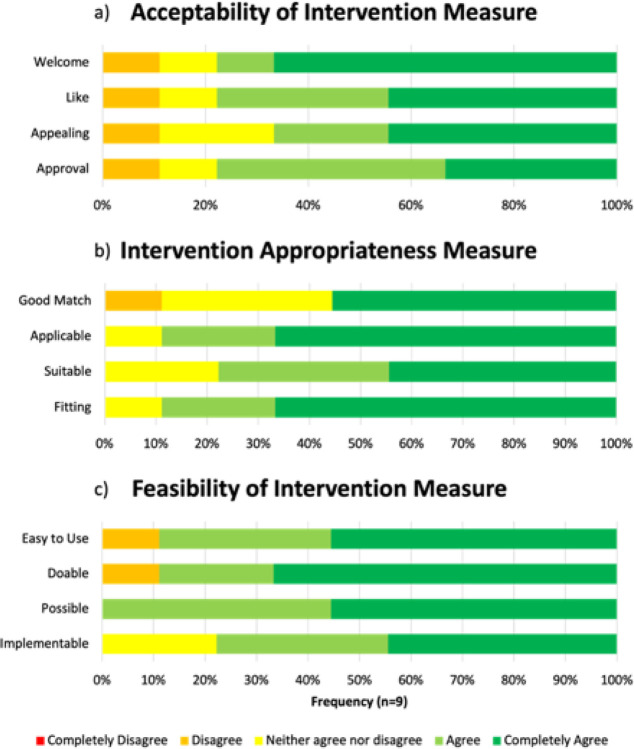
Distribution of participant responses on item-level ratings of the video series’ acceptability (Panel A), appropriateness (Panel B), and feasibility (Panel C).

Of the 6-month survey responders, nine participants reported viewing the online videos and completed the acceptability, appropriateness, and feasibility measures. Overall, the mean acceptability, appropriateness, and feasibility scores were 4.1 (SD 1.0), 4.3 (SD 0.8), and 4.4 (SD 0.7) out of 5, respectfully. Most participants completely agreed or agreed that the online videos were acceptable, appropriate, and feasible (Fig. 2). Only one participant disagreed with the acceptability statements that the videos were ‘welcoming’, that they ‘liked’ the videos, that the videos were ‘appealing’, and they ‘approved’ of the videos. Notably, no participant strongly disagreed that the videos were acceptable appropriate, or feasible.

### PROMIS Sexual Function and Satisfaction

**Figure 3:**
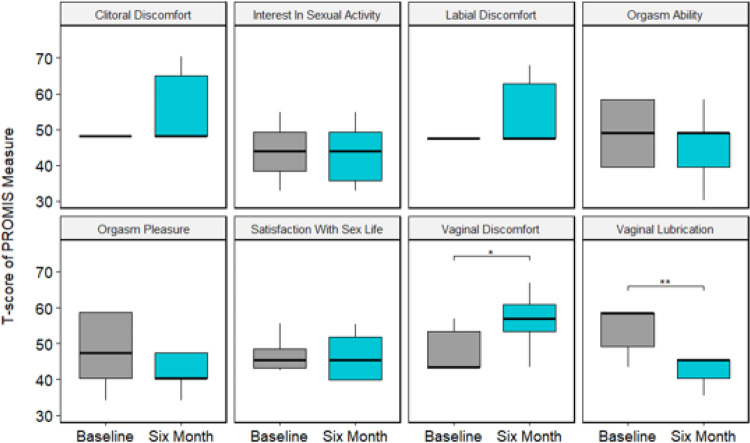
PROMIS Brief Female Sexual Function scores at baseline and 6 months (*p<0.05, **p<0.01). Worsening function is indicated by negative (−) change scores in domains of interest in sexual activity, vaginal lubrication, orgasm ability, orgasm pleasure, and satisfaction with sex life; and by positive (+) change scores in subdomains of vaginal discomfort, labial discomfort, and clitoral discomfort.

Eleven participants completed the PROMIS BRIEF Female Sexual Function and Satisfaction measure at baseline and 6 months. Two participants indicated no sexual activity during the past 30 days, excluding them from answering the remaining domain-specific questions. Changes in sexual function were observed across all symptom domains at 6-months, with mean change scores reflecting possible worsening sexual function from baseline to 6-month follow up (Fig. 3, Supplemental Table 1). Across sexual function subdomains, decreased vaginal lubrication (T-score change = −8.5, SD = 7.3, p < 0.01) and increased vaginal discomfort (T-score change = + 6.5, SD = 7.2, p-value = 0.03) were the most pronounced with changes in sexual activity, labial discomfort, clitoral discomfort, orgasm ability, orgasm pleasure, and satisfaction with sex life not reaching statistical significance.

### Free Response Answers

All participants shared a range of insights when asked the stand-alone question, “What did you learn from the videos?” (Table 3). Five participants reported on content-specific learnings including new knowledge gained about expected physical changes due to cancer and its associated treatment. One participant focused on the structure and delivery of the content and felt they were too “clunky, repetitive, and too short”. Finally, three participants felt the scope was too narrow, ultimately reporting a desire for additional detail or reporting that the content in the videos was similar to their own online research.

## Conclusions

In this pilot study, a sample of newly diagnosed breast cancer patients found our online sexual health educational video series to be highly acceptable, appropriate, and feasible as an educational intervention. This study represents an important proof of concept for this novel, patient-centered, video-based intervention. While limited by a small sample size, this exploratory work provides valuable insights into patients’ needs, preferences, and ideas for video optimization.

To our knowledge, this is the first study to expand on the use of video-based education, developed in partnership with breast cancer survivors, to provide resources for breast cancer patients inclusive of treatment-related sexual health changes. The online accessibility of the videos supports the feasibility as it allows patients to privately access the videos in a time and place that are most convenient to them. With the growth and accessibility of the internet, many patients turn towards resources they can access through their web browsers and social media. TikTok, a social media platform for creating, sharing, and watching short-form videos, is one such online place where patients may seek information about breast cancer and breast cancer surgery [37, 38]. However, studies to characterize the information about breast cancer on TikTok have shown that while videos have high engagement, the videos are not commonly created by healthcare professionals, are fragmented, and have poor content and information quality [38, 39]. Conversely, existing video-based education developed by providers has been demonstrated to be feasible [40], improve breast cancer patient knowledge [40, 41], and reduce post-operative anxiety and increase comfort [42]. However, these video series often focus on general breast cancer content and lack sexual health specific information [40, 42]. The online video series in this study mixes the style of TikTok videos (e.g., short in length with real patient stories) with evidence-based information developed by subject matter experts [28]. The videos were an average length of 1.4 minutes; thus, we hypothesized that this would be welcomed by the patients who are often overwhelmed with the volume of information to learn after a diagnosis. Surprisingly, while nearly all participants felt the videos were acceptable, appropriate, and feasible via Likert responses, when asked what they learned from the videos several participants felt the videos were too short and wanted greater detail. Future semi-structured interviews with study participants aim to qualitatively explore the individual use of the videos and identify specific topics with insufficient detail. Additionally, testing of the videos among a larger patient population is also necessary to identify more perspectives on the content, scope, and delivery of the video series.

In the absence of available online information, patients must rely on their healthcare providers, family, and friends for resources and information. Using focus groups, Huynh et al., found that many breast cancer survivors never received sexual health education from their oncology team or other medical providers. When they did, the education focused on reproductive health or menopausal symptoms [6]. In another study assessing factors affecting willingness to discuss symptoms with providers, researchers found that many patients experience barriers such as discomfort bringing sexual health up, a belief that their symptoms are untreatable, and difficulty finding a provider to take ownership of dealing and assisting with their sexual health concerns [43]. Clinicians also report significant barriers to discussing sexual health including time, conflicting priorities, and discomfort discussing sexual health [12, 15]. To improve sexual health discussions, a multifaceted approach should be taken that addresses both patients and providers. One potential way to better support patients is to teach them how to discuss their sexual health with their providers. For example, a patient-facing multimedia intervention consisting of a 20-minute video slideshow and workbook led to more women raising sexual health topics during their clinic visits [20]. While the online video series in this study does not directly coach patients in how to communicate with providers about their sexual health, it may have helped to normalize this topic as part of cancer care, which in turn may lead to improved self-efficacy and confidence in broaching it when symptoms arise. Importantly, this normalization of sexual health has been previously shown to support patient comfort in raising the topic [43]. As such, future studies should investigate specific cognitive (e.g., self-efficacy) and behavioral (e.g., clinical communication) outcomes of the video series.

Regarding the impact of the video series on sexual health outcomes, this study was exploratory in nature. Given that more than 70% of the 6-month responders were undergoing treatment with hormonereceptor blockers at the time of their survey, our findings of the wide range of changes across sexual function subdomains is not surprising. Among breast cancer patients, these are the most common sexual health symptoms with vaginal dryness and pain with intercourse occurring in up to 85% and 87% of patients, respectively [10, 25]. As many breast cancer patients are undergoing treatment for longer than our six-month study, it’s impossible to directly assess the impact of the video series on these sexual health changes or if access to the videos led patients to obtain treatment resources sooner than patients who may not have had the same education. The next phase of study will examine the impacts of the videos over a longer period, which will allow for further exploration. Additionally, future studies should include a control group to objectively evaluate the effect the video series has on sexual function.

Limitations of this study include a small sample size and short timeframe. Participants who completed the six-month survey may have been different than the participants who did not complete the survey in areas we did not measure, such as participants being more interested in sexual health education. However, when comparing the demographic and clinical characteristics of the 6-month responders to the non-responders, we did not find any statistically significant differences. While a small number of participants withdrew from the study due to feeling overburdened (Supplemental Fig. 1), many participants never responded to the 6-month survey despite repeated attempts to engage. It’s possible that more participants decided they did not want to continue in the study after realizing how many videos were on the website. Others may have logged in and watched a few videos and then forgotten about them as their treatment progressed despite monthly emailed reminders. We purposefully did not give participants specific instructions on how to engage with the videos other than to watch the ones they were interested in, but this may be one way to reduce this barrier. For example, perhaps the videos are “prescribed” to the patient during their cancer care. A prescription would personalize the library of content for an individual based on their breast cancer and other health needs while also introducing the possibility of ensuring that patients receive the right information at the right time. It is also possible that participants engaged with the videos but neglected to complete the 6-month survey. In fact, an unpublished group-level analysis of engagement with the video website demonstrated that over 6 months, there was 130 unique sessions with total video watch time of 524 minutes and an average of 8 minutes per session [44]. Another limitation is the availability of the online videos in English-only which leads to disparities in care for non-English speaking patients who must rely on standard education and cannot utilize this novel resource. The videos are currently being translated into Spanish, but future work should include expanding access across other languages as well. Finally, this study wasn’t powered to detect statistical significance but was intended to identify implementation challenges and inform future study design. Nevertheless, among the women who did watch the video series, responses were overall positive.

In conclusion, this pilot study demonstrates the acceptability, appropriateness, and feasibility of a novel sexual health education video series for female breast cancer patients with Stage I-III disease. This video series fills a unique gap in patient care, and with further iterations and development has the potential to significantly advance the sexual health-resources available to breast cancer patients and survivors.

## Supplementary Material

Supplementary Files

This is a list of supplementary files associated with this preprint. Click to download.

- SupplementalFiguresSexualHealthVideoSeriesSCC.docx

## Figures and Tables

**Table 1:** Patient demographic characteristics and sexual activity in the past 6 months.

	Overall n = 28	6-month non-responder n = 17	6-month responder n = 11	p-value
Age, Mean (SD)	47.9 (8.0)	47.8 (7.1)	48.1 (9.7)	0.93
Ethnicity % (N)				0.39
Not Hispanic/Latina or of Spanish origin	96.4% (27)	100.0% (17)	90.9% (10)	
Hispanic/Latina/Spanish origin	3.6% (1)	0.0% (0)	9.1% (1)	
Race %(N)				>0.99
American Indian or Alaska	3.6% (1)	5.9% (1)	0.0% (0)	
Caucasian/White	85.7% (24)	82.4% (14)	90.9% (10)	
Other (specify)	3.6% (1)	5.9% (1)	0.0% (0)	
Prefer not to answer	7.1% (2)	5.9% (1)	9.1% (1)	
Sexual Orientation % (N)				0.34
Heterosexual or straight	89.3% (25)	94.1% (16)	81.8% (9)	
Lesbian, gay, homosexual, or same- gender loving	3.6% (1)	0.0% (0)	9.1% (1)	
Bisexual	3.6% (1)	0.0% (0)	9.1% (1)	
Prefer not to answer	3.6% (1)	5.9% (1)	0.0% (0)	
Gender Identity % (N)				0.39
Female	96.4% (27)	100.0% (17)	90.9% (10)	
Unknown	3.6% (1)	0.0% (0)	9.1% (1)	
Relationship status % (N)				>0.99
Single/ not dating	3.6% (1)	5.9% (1)	0.0% (0)	
Dating/ boyfriend/ girlfriend	10.7% (3)	11.8% (2)	9.1% (1)	
Partnered	25.0% (7)	23.5% (4)	27.3% (3)	
Married	60.7% (17)	58.8% (10)	63.6% (7)	
Sexual Partner Gender in the Past 6 months *%* (N)				0.07
Female	7.1% (2)	0.0% (0)	18.2% (2)	
Male	75.0% (21)	76.5% (13)	72.7% (8)	
Other	7.1% (2)	11.8% (2)	0.0% (0)	
Female and Male	7.1% (2)	11.8% (2)	0.0% (0)	
Unknown	3.6% (1)	0.0% (0)	9.1% (1)	
Sexual activity in the past 6 months % (N)				0.26
None/ abstinence / prefer not to answer	10.7% (3)	17.6% (3)	0.0% (0)	
Any sexual activity	89.3% (25)	82.4% (14)	100.0% (11)	

**Table 2:** Patient clinical characteristics including breast cancer treatment received over the course of the study. (ER = estrogen receptor, PR = progesterone receptor, AIs = aromatase inhibitors)

	Overall n = 28	6-month non-responder n = 17	6-month responder n = 11	p-value
Breast cancer diagnosis, % (N)				0.47
Invasive lobular carcinoma	7.1% (2)	11.8% (2)	0.0% (0)	
Invasive ductal carcinoma	82.1% (23)	82.4% (14)	81.8% (9)	
Other	10.7% (3)	5.9% (1)	18.2% (2)	
Clinical anatomic stage, % (N)				0.84
Stage I	67.9% (19)	70.6% (12)	63.6% (7)	
Stage II	21.4% (6)	17.6% (3)	27.3% (3)	
Stage III	10.7% (3)	11.8% (2)	9.1% (1)	
Hormone Receptor Status, % (N)				>0.99
ER/PR+, HER2+	14.3% (4)	11.8% (2)	18.2% (2)	
ER/PR +, HER2−	75.0% (21)	76.5% (13)	72.7% (8)	
ER/PR−, HER2−	10.7% (3)	11.8% (2)	9.1% (1)	
Surgery, % (N)				0.33
Lumpectomy	50.0% (14)	58.8% (10)	36.4% (4)	
Mastectomy	46.4% (13)	41.2% (7)	54.5% (6)	
None	3.6% (1)	0.0% (0)	9.1% (1)	
Chemotherapy, % (N)				>0.99
Neoadjuvant chemotherapy	25.0% (7)	23.5% (4)	27.3% (3)	
Adjuvant chemotherapy	14.3% (4)	17.6% (3)	9.1% (1)	
Both	10.7% (3)	11.8% (2)	9.1% (1)	
None	50.0% (14)	47.1% (8)	54.5% (6)	
Endocrine therapy (Tamoxifen, AIs), % (N)	57.1% (16)	47.1% (8)	72.7% (8)	0.25
Radiation therapy, *%* (N)	64.3% (18)	64.7% (11)	63.6% (7)	>0.99

**Table 3:** Participant comments regarding what they learned after six months of using the educational video series. The open-ended question asked participants, **“What did you learn from the videos?”**.

	Participant Quote
**Content-specific**	a) “A better idea of what to expect for bodily changes due to cancer and some upcoming treatment. I never even thought of how cancer could affect sexual health so it was nice hearing from former patients.” (Participant #1) b) “I learned about sexual health during breast cancer. Also, what procedures would be.” (Participant #4) c) “I learned about what to expect with both radiation and tamoxifen.” (Participant #5) d) “Helpful overview of breast cancer before and after surgery.” (Participant #6) e) “I learned about some of the common side effects to treatments as well as what the surgery options were.” (Participant #9)
**Structure & Delivery**	a) “I only watched a few videos about mastectomy, but found the videos kind of clunky, repetitive and too short so I didn’t watch more videos.” (Participant #8)
**Scope**	a) “Most of the information I learned from my doctor. These videos are nice and short, but didn’t seem to go into very much detail.” (Participant #2) b) “The only one that was helpful was the one on the various mastectomy choices and expectations. The others were too basic and vague.” (Participant #3) c) “Most of the information I already know or did not pertain to me. But that may be because I did a lot of research prior to watching the videos.” (Participant #7)
